# Substrate Channel Flexibility in *Pseudomonas aeruginosa* MurB Accommodates Two Distinct Substrates

**DOI:** 10.1371/journal.pone.0066936

**Published:** 2013-06-21

**Authors:** Ming Wei Chen, Bernhard Lohkamp, Robert Schnell, Julien Lescar, Gunter Schneider

**Affiliations:** 1 Department of Medical Biochemistry and Biophysics, Karolinska Institutet, Stockholm, Sweden; 2 School of Biological Sciences, Nanyang Technological University, Singapore, Singapore; Institute of Enzymology of the Hungarian Academy of Science, Hungary

## Abstract

Biosynthesis of UDP-*N*-acetylmuramic acid in bacteria is a committed step towards peptidoglycan production. In an NADPH- and FAD-dependent reaction, the UDP-*N*-acetylglucosamine-enolpyruvate reductase (MurB) reduces UDP-*N*-acetylglucosamine-enolpyruvate to UDP-*N*-acetylmuramic acid. We determined the three-dimensional structures of the ternary complex of *Pseudomonas aeruginosa* MurB with FAD and NADP^+^ in two crystal forms to resolutions of 2.2 and 2.1 Å, respectively, to investigate the structural basis of the first half-reaction, hydride transfer from NADPH to FAD. The nicotinamide ring of NADP^+^ stacks against the *si* face of the isoalloxazine ring of FAD, suggesting an unusual mode of hydride transfer to flavin. Comparison with the structure of the *Escherichia coli* MurB complex with UDP-*N*-acetylglucosamine-enolpyruvate shows that both substrates share the binding site located between two lobes of the substrate-binding domain III, consistent with a ping pong mechanism with sequential substrate binding. The nicotinamide and the enolpyruvyl moieties are strikingly well-aligned upon superimposition, both positioned for hydride transfer to and from FAD. However, flexibility of the substrate channel allows the non-reactive parts of the two substrates to bind in different conformations. A potassium ion in the active site may assist in substrate orientation and binding. These structural models should help in structure-aided drug design against MurB, which is essential for cell wall biogenesis and hence bacterial survival.

## Introduction

Peptidoglycan (PG) is a vital component of the cell wall in most prokaryotic organisms. The high molecular weight polymer provides osmotic stability and defines the size and shape of bacterial cells [1]. The cell wall PG of bacteria comprises a glycan chain of alternating N-acetyl-glucosamine and N-acetyl-muramic acid units, which are linked via peptide crosslinks. In Gram-negative bacteria, the formation of the high molecular weight PG polymer takes place in the periplasmic space, whereas the disaccharide-pentapeptide building blocks of PG are produced in the bacterial cytoplasm, linked to polyprenyl lipid chains and attached to the cytoplasmic membrane [2]. Following translocation to the periplasmic side of the membrane, the PG building blocks are accessible to penicillin binding proteins, the enzymes responsible for the polymerization of PG units [2]–[4]. The cytoplasmic biosynthetic pathway leading to these PG-units involves ten enzymes [3], [4]. UDP-*N*-acetylglucosamine (UNAG) is first synthesized from fructose-6-phosphate, acetyl-CoA and uridyl triphosphate. To produce the glycan building block UDP-*N*-acetylmuramic acid (UNAM), an enolpyruvyl group is transferred from phosphoenolpyruvate to UNAG by the UNAG enolpyruvyl transferase, MurA [5]. The resulting UDP-*N*-acetylglucosamine enolpyruvate (UNAGEP) is reduced by the UNAGEP reductase, MurB [6], to generate UNAM. Then, the pentapeptide stem is added in consecutive steps to the lactoyl moiety of UNAM [3], [4].

**Figure 1:**
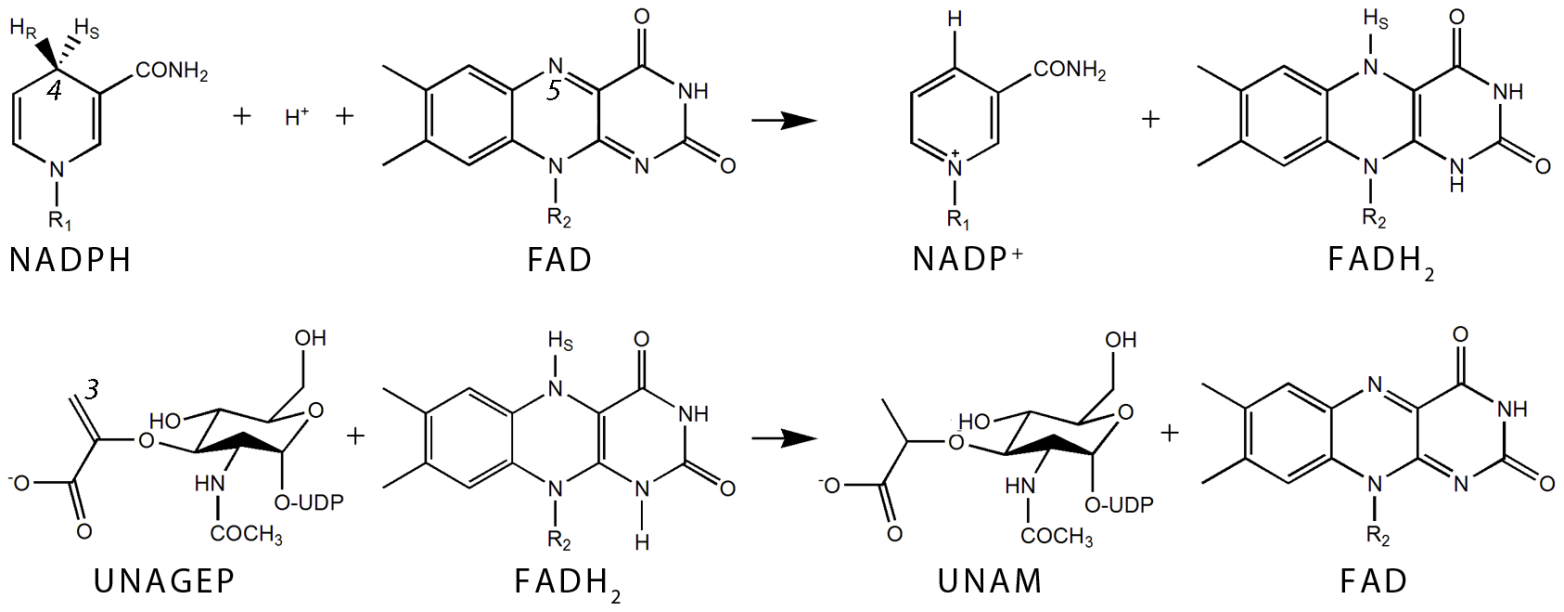
MurB-catalyzed FAD- and NADPH-dependent reduction of UNAGEP to UNAM. In the first half reaction (upper panel) the 4-*pro*-S hydrogen and two electrons from the reduced nicotinamide C4 atom are transferred to the N5 atom of the isoalloxazine resulting in NADP^+^ and reduced FAD. In the second half reaction (lower panel) the hydride is transferred to the C3 atom of the UNAGEP enolpyruvyl group, reducing the enolpyruvyl to a lactoyl group and thus converting UNAGEP to UNAM.

All MurB proteins known to date are monomeric flavoenzymes that bind strongly a stoichiometric amount of flavin adenine dinucleotide (FAD). Extensive kinetic studies on *Escherichia coli* MurB (EcMurB) revealed that nicotinamide adenine dinucleotide phosphate (NADPH), but not nicotinamide adenine dinucleotide (NADH), is utilized as the cosubstrate [6], [7]. The catalytic cycle can be divided into two half reactions and follows the scheme of a ping-pong bi bi mechanism (Fig 1). NADPH transfers the 4-*pro*-S hydrogen to the isoalloxazine N5 atom during the first half-reaction [3], [6] and dissociates from the enzyme. This step is followed by the binding of UNAGEP and the hydride transfer from FADH_2_ to the vinyl ether of UNAGEP. The complete reaction thus converts NADPH and UNAGEP to NADP^+^ and UNAM, respectively. Crystal structures have been determined for MurB enzymes of *E. coli* (EcMurB, PDB code 1MBT) [8], *Staphylococcus aureus* (SaMurB, 1HSK) [9], *Thermus caldophilus* (TcMurB, 2GQT) [10], *Listeria monocytogenes* (LmMurB, 3TX1) and *Vibrio cholerae* (VcMurB, 3I99), which share a common three-domain architecture. Domains I and II form the FAD-binding module that is conserved in a wide range of oxidoreductases including cytokinin dehydrogenase [11] and the broad-specificity vanillyl-alcohol oxidase [12]. The substrate-binding domain III is unique but has two variations that divide MurB enzymes into two types. Type I MurB, exemplified by the prototypical EcMurB, contains a tyrosine loop and a protruding βαββ fold. The type II enzymes lack these features on domain III and are exemplified by SaMurB and TcMurB. Furthermore, type IIa MurB utilizes a serine residue as a proton donor to the UNAGEP carbanion intermediate [9], [13], [14], while the type IIb group employs cysteine instead [10].

In the crystal structure of EcMurB in complex with UNAGEP (PDB code 2MBR), the enolpyruvyl moiety of UNAGEP packs against the *si* face of the isoalloxazine ring of FAD, bringing the ultimate hydride acceptor atom C3 of the enolpyruvyl (C3e) close to the isoalloxazine N5 atom [15]. However, no experimental structure of the MurB-NADP(H) complex has been reported to date. Based on the comparison between substrate-free and UNAGEP-bound EcMurB, NADPH was proposed to bind in the UNAGEP site or in a perpendicular channel close to the C-terminus of the enzyme, both resulting in the unusual binding of nicotinamide to the *si* face of isoalloxazine [8]. Alternatively, the nicotinamide ring could approach the FAD *re* face by rearranging a loop at the domain-II-domain-III interface, thus allowing NADPH to bind perpendicular to the UNAGEP site [15]. NMR studies of the EcMurB-NADP^+^ complex suggested that NADPH and UNAGEP share at least a binding subsite [16], [17]. Moreover, NADP^+^ can reverse the reduction of FAD, bind to both reduced and oxidized forms of MurB with high affinity [18], and inhibit the reaction non-competitively with regard to both NADPH and UNAGEP [7].

Due to the essentiality of peptidoglycans in most pathogenic bacteria, PG biosynthetic enzymes are the target of several antibiotics. Substrate-bound structures of MurB are therefore of great value in rational design of improved and novel inhibitors of peptidoglycan biosynthesis. As part of the effort to elucidate the structures of important *Pseudomonas aeruginosa* drug targets [19], we produced and crystallized *P. aeruginosa* MurB (PaMurB; gene locus PA2977). Here, we present the crystal structure of NADP^+^-bound PaMurB, which establishes the co-localization of the NADPH and UNAGEP binding sites and supports a ping pong reaction mechanism with sequential substrate binding. The crystal structure of the ternary complex of PaMurB-FAD-NADP^+^ provides a snapshot of the first step of the catalytic cycle in this enzyme class, implying an unusual hydride transfer from nicotinamide to the *si* face of flavin.

## Materials and Methods

### Production and purification of PaMurB

The coding sequence for PaMurB (PA2977 gene) was amplified by PCR using the *P. aeruginosa* PAO1 genomic DNA template (ATCC 47085) and cloned into the pNIC28-Bsa4 vector (GenBank Accession No. EF198106) using the ligation-independent cloning method. The expression construct encodes a fusion protein with an N-terminal His_6_-tag (H_2_N-MHHHHHHSSGVDLGTENLYFQ*SM) and a TEV protease cleavage site (indicated by *). The construct was expressed in *E. coli* BL21(DE3) in 2 L LB media containing 50 µg/mL kanamycin inoculated with 20 mL of an overnight seed culture. The culture was grown with vigorous shaking to OD_600_ of 0.7, chilled on ice, induced with 0.2 mM IPTG, and grown at 18°C for 20 hours. Cells were harvested by centrifugation at 3300 g and resuspended in lysis buffer (50 mM Tris-HCl pH 7.5, 0.5 M NaCl, 5% v/v glycerol). Lysate was produced by ultrasonication on ice (8 cycles of 30 s), clarified by centrifugation at 45000 g and subsequently filtered. The lysate was incubated with 1 mL Ni-NTA resin (Qiagen) in the presence of 10 mM imidazole for one hour with gentle agitation. The protein was eluted with buffer containing 200 mM imidazole. Subsequently the buffer was exchanged to 25 mM Tris-HCl pH 8.0 containing 150 mM NaCl using a PD-10 column (GE Healthcare). For His_6_-tag removal 2 mM DTT and His_6_-tagged TEV protease (at 1∶200 protease-to-PaMurB mass ratio) were added and incubated at 20°C for 16 hours. A subsequent Ni-NTA affinity step removed uncleaved protein and the His_6_-tagged TEV protease. All following steps and experiments are based on tag-free PaMurB carrying one extra serine residue in the N-terminal position. Purified PaMurB was concentrated and loaded on an S200 prep grade gel-filtration column (GE Healthcare) equilibrated with a buffer containing 25 mM Tris-HCl pH 8.0 and 150 mM NaCl, and eluted as a single peak. Pure fractions were pooled and concentrated with Vivaspin microconcentrator units (Sartorius) to 25 mg/mL, flash-frozen in liquid nitrogen, and stored at −80°C.

### Crystallization of PaMurB

PaMurB at 25 mg/mL concentration with 2 mM unbuffered NADPH (Sigma-Aldrich) or 2 mM Tris-(pH 8.0)-buffered NADP^+^ sodium salt (Sigma-Aldrich) was used to set up 96-well format crystallization trials. A Mosquito crystallization robot (TTP LabTech Ltd, Melbourn UK) and commercially available crystallization screening reagents (JSCG+ and PACT from Qiagen, SaltRX from Hampton Research) were used. Two different crystal forms were obtained and used for X-ray diffraction data collection. During crystal optimization, crystal form A was obtained by mixing 1 µL of the protein solution (25 mg/ml PaMurB, 2 mM unbuffered NADPH) with 1 µL of precipitant (0.1 M bis-tris propane pH 7.0, 0.2 M sodium potassium tartrate, 15% w/v PEG 3350), followed by equilibration against 0.5 mL of reservoir solution via vapour diffusion. Crystals from these drops were cryoprotected with a solution containing additional 15% w/v PEG3350 and 2 mM NADPH before flash-freezing. Crystal form B was obtained from a condition containing 40 mM potassium phosphate, 20% v/v glycerol, 16% w/v PEG 8000 and 2 mM Tris-buffered NADP^+^ sodium salt and were frozen directly without addition of a cryoprotectant.

### Data collection and processing

X-ray diffraction data for crystal forms A and B were collected at the European Synchrotron Radiation Facility (ESRF) beamline stations ID14-4 and ID14-1, respectively. Data were indexed and integrated with iMosflm [20], and subsequently scaled using SCALA [21] (for form A) and AIMLESS [22] (for form B) from the CCP4 package [23]. Form A crystals are of space group C2, with cell dimensions a = 153.7 Å, b = 154.3 Å, c = 64.5 Å and β = 102.3°, while form B crystals belong to space group P6_1_ with cell parameters a = 88.7 Å and c = 100.2 Å. We could not detect any signs of twinning in these crystals using the twin tests [24] implemented in CTRUNCATE. Crystals of form A showed a somewhat streaky and anisotropic diffraction pattern, reflected in a relatively high R_merge_ value. The X-ray diffraction data statistics are given in Table 1.

**Table 1:** Data collection and structure refinement statistics.

Data set	Crystal form A	Crystal form B
*Data collection statistics*		
Beam line	ID14-4, ESRF	ID14-1, ESRF
Wavelength (Å)	0.9762	0.9334
Resolution (Å)	58.38–2.23 (2.35–2.23)	50.11–2.10 (2.16–2.10)
Space group	C2	P6_1_
Cell parameters a/b/c (Å)	153.7, 154.3, 64.5	88.7, 88.7, 100.2
α/β/γ (°)	90.0, 102.3, 90.0	90.0, 90.0, 120.0
Unique reflections	69794 (9862)	26203 (2179)
Redundancy	3.0 (2.6)	7.6 (7.6)
I/σ	3.8 (1.6)	13.1 (2.9)
Completeness (%)	98.2 (95.7)	100.0 (100.0)
R_merge_	0.215 (0.686)	0.109 (0.666)
R_pim_	0.143 (0.507)	0.064 (0.390)
Wilson B factor (Å^2^)	20.0	21.6
*Refinement statistics*		
Data range (Å)	54.33–2.23 (2.29–2.23)	44.39–2.10 (2.16–2.10)
Used reflections	66255	24851
R_work_	0.222 (0.348)	0.180 (0.272)
R_free_	0.258 (0.367)	0.236 (0.320)
No of monomers/ASU	4	1
No. of protein atoms	10580	2732
No. of FAD molecules	4	1
No. of NADP^+^ molecules	4	1
No. of water molecules	340	164
No. of K^+^	4	-
*Average B-factors (Å^2^)*		
Protein	33.1	42.5
FAD	14.8	25.4
NADP^+^	25.2	30.0
Water molecules	27.1	41.0
K^+^ ions	19.0	-
*R.m.s. deviations*		
Rmsd bond length (Å)	0.014	0.019
Rmsd bond angles (°)	1.700	2.125
*Ramachandran plot (% residues)*		
Allowed	1319 (98.1%)	322 (95.8%)
Generously allowed	25 (1.9%)	13 (3.9%)
Disallowed	0 (0.0%)	1 (0.3%)

Values in parentheses are for the highest-resolution shell, ASU: Asymmetric unit.

### Structure solution, refinement and analysis

The structure of PaMurB was solved by molecular replacement using MOLREP [24] with the atomic coordinates of the complex of EcMurB with UNAGEP (PDB code 2MBR), stripped of all ligands and water molecules as search model. Molecular replacement solutions for data of crystal forms A and B were acquired independently with the search model, and contained four and one molecule(s) per asymmetric unit, respectively (Table 1). Models were built by alternating rounds of model-building in WinCoot [25] and refinement in REFMAC5 [26]. The protein models of crystal forms A and B were refined to 2.23 and 2.10 Å, respectively. Where one model displayed ambiguous electron density, model-building was aided by referring to the other model. A TLS analysis was performed for both protein models using the TLSMD server [27], [28]. The results were used for restrained refinement with TLS in REFMAC5, with 7 segments for crystal form B, resulting in an improvement of R_work_/R_free_ of 2.0%/2.5%. Two TLS segments per monomer were employed during initial refinement of crystal form A. Space groups were validated by using ZANUDA from the CCP4 suite [23]. The quality of the models was assessed using MolProbity [29].

**Figure 2:**
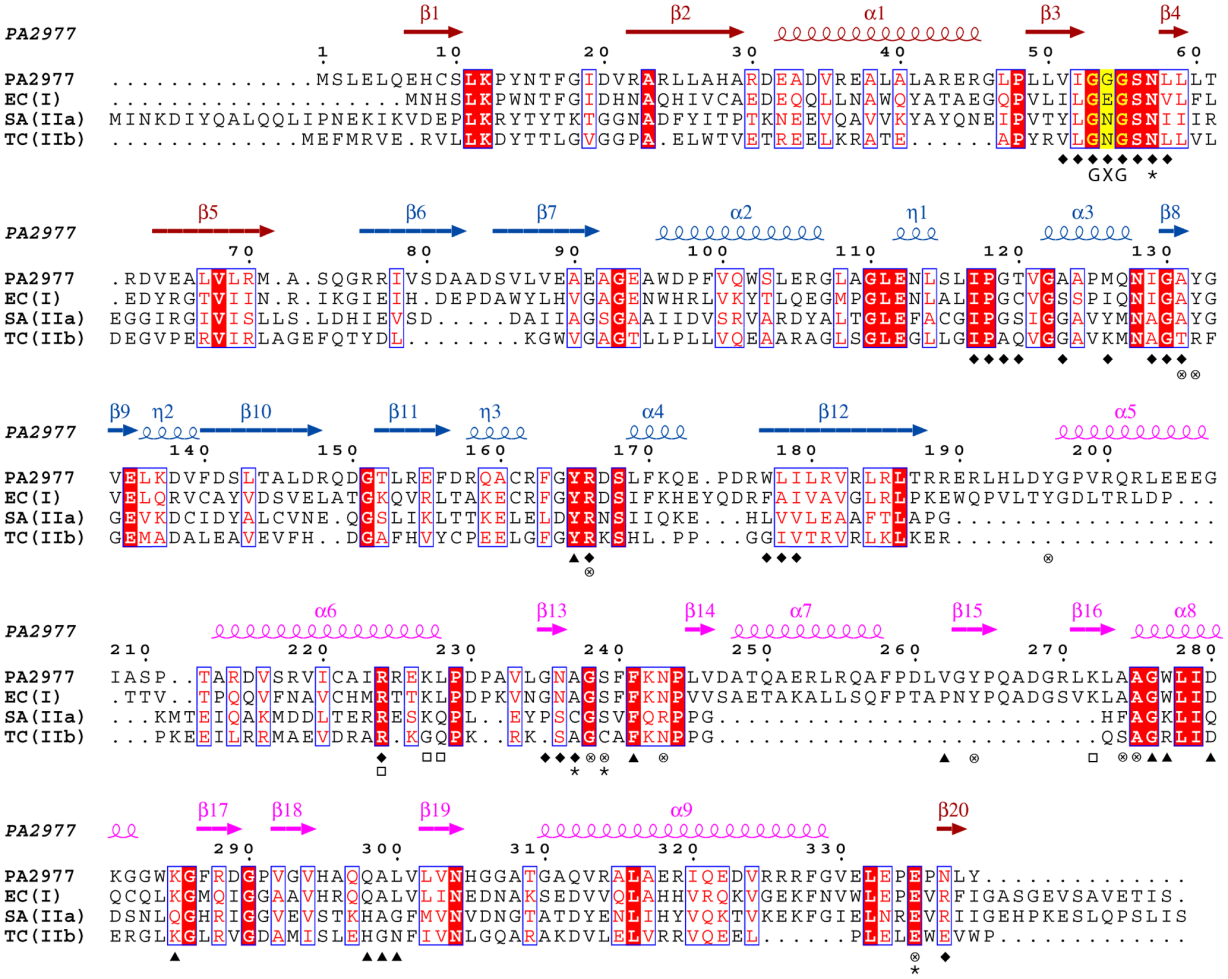
Structure-based sequence alignment of PaMurB against three MurB enzymes with experimentally determined crystal structures. Each of the three enzymes represents one type of MurB. Annotations of secondary structure are based on PaMurB and are colored red for FAD-binding domain I, blue for domain II and pink for substrate-binding domain III. Strictly conserved residues are shaded in red and conserved regions are boxed; the GXG motif important for FAD-binding is indicated and shaded in yellow. The sequences compared are *Pseudomonas aeruginosa* MurB (PA2977), *Escherichia coli* MurB (EC(I), type I, PDB code 2MBR), *Staphylococcus aureus* MurB (SA(IIa), type IIa, PDB code 1HSK), and *Thermus caldophilus* MurB (TC(IIb), type IIb, PDB code 2GQT). Residues involved in cofactor- and substrate-binding are indicated: ⧫, residues that interact with FAD in both PaMurB and EcMurB; ▴, equivalent residues that interact with UNAGEP in EcMurB; □, residues that interact with NADP^+^;⊗, residues that interact with both UNAGEP and NADP^+^; ✶, residues that coordinate the putative catalytic metal ion.

Superimposition of structural models was performed in WinCoot; the SSM [30] method was used for superimposing protein backbones while the LSQ method was used when ligand-based structural alignment was required. Structure-aided multiple sequence alignment, secondary structure assignment and generation of Figure 2 was performed using STRAP [31], STRIDE [32] and ESPript [33], respectively. The ligand-protein interaction maps were generated by LIGPLOT+ [34] and manually enhanced. The remaining figures were made with PyMOL (www.pymol.org). The crystallographic data and models for crystal form A and B were deposited with the Protein Data Bank, accession codes 4JAY and 4JB1, respectively.

## Results

### Structure determination and quality of the models

The crystal structure of PaMurB in complex with NADP^+^ was determined in two different crystal forms by molecular replacement. Crystal form A belongs to the space group C2 with four molecules per asymmetric unit, while crystal form B belongs to P6_1_ with one molecule per asymmetric unit; the solutions are in agreement with solvent content analysis by using Matthews coefficient [35]. The models were refined to R_work_/R_free_ of 22.3%/25.8% and 18.2%/23.4% using data to the resolution of 2.23 and 2.10 Å, respectively. Ligands and the polypeptide chains are well-defined except the first two residues of the N-termini (Ser-0, Met-1). Also for crystal form A, the resulting electron density maps were of good quality, most likely due to the presence of four-fold non-crystallographic symmetry. Statistics for the refinement and the structural models are provided in Table 1. Ala-83, located on the β6-β7 turn, is a Ramachandran plot outlier in crystal form B with well-defined electron density, possibly because of crystal contacts.

### PaMurB is a type I UNAGEP reductase

Based on the sequence alignment, PaMurB is expected to be a type I UNAGEP reductase, similar to EcMurB (Fig 2). Size-exclusion chromatography indicated a monomeric state in solution (Fig. S1). Consistent with this finding, an analysis of oligomeric assembly by the PISA server [36] indicated that PaMurB exists also as a monomer in both crystal forms. The two crystal structures are identical in protein and ligand conformations, except three loops involved in crystal contacts on one side of the protein (β6-β7, β12-α5 and α5-α6) with an overall root-mean-squared difference, rmsd, of 0.8 Å, based on 336 Cα atoms. In the following results and figures are based on crystal form B unless otherwise noted.

**Figure 3:**
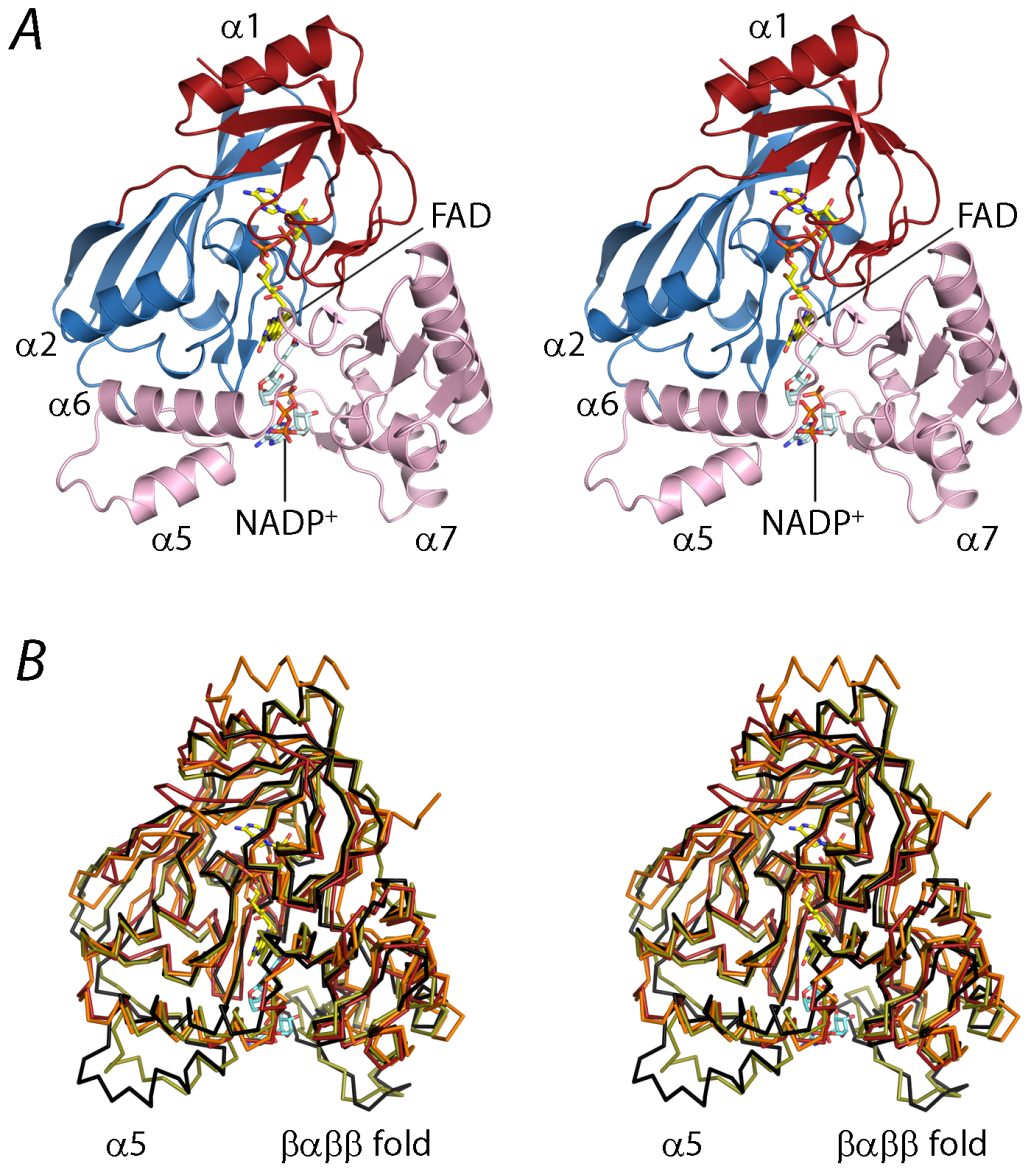
Overall structure of the ternary complex of PaMurB with FAD and NADP^+^. **A**. Stereo view of the crystal structure of PaMurB with bound FAD and NADP^+^. The enzyme is shown in cartoon representation and comprises FAD-binding domain I (red) and domain II (blue), and the substrate-binding domain III (pink). FAD and NADP^+^ are shown as yellow and cyan stick models, respectively. NADP^+^ occupies the channel between the two lobes of domain III (in this view: left, lobe 1; right, lobe 2). Relevant secondary structure elements are labeled. **B**. Stereo view of the superimposition of the Cα traces of EcMurB (olive green), SaMurB (orange) and TcMurB (red) against PaMurB (black). PaMurB and EcMurB display highly similar structures in their respective NADP- and UNAGEP-bound complexes. In domain III, type II MurB enzymes lack the tyrosine loop preceding helices α4 and α5, as well as the protruding βαββ fold on lobe 2.

PaMurB consists of three domains: the FAD-binding domains I (aa 1–75 and 336–339) and II (aa 76–191), and the substrate-binding domain III (aa 192–335) (Fig 3A). The structural framework of the FAD-binding module is provided by two β-sheets (β1-β2-β5-β3 and β6-β7-β12-β10-β11) packed against flanking helices α1 and α2, respectively. The buried cofactor binding pocket is formed by the GXG motif on the β3-β4 strand-loop-strand (Fig 2) and the C-terminal strand β20 contributed by domain I, and the β7-α3 loop, the η1- α3- β8 region, and α4- β12 from domain II. The substrate-binding domain III is adjacent to the FAD pocket and can be visualized as two lobes (Fig 3). Lobe 1 consists of the linker originating from domain II and helices α5-α6, interacting with domain II. Lobe 2 is adjacent to domain I and features the β13-β19-β18-β17 sheet and the prominent β14-α7-β15-β16 outcrop. The substrate binding site, located between the lobes, is more shallow and solvent-accessible than the cofactor site.

The structures of substrate-bound PaMurB and EcMurB (PDB code 2MBR) are very similar (rmsd 1.3 Å based on 321 Cα atoms) (Fig. 3B), consistent with the moderately high sequence identity of 45% (Fig. 2). Compared to EcMurB, PaMurB has a longer N-terminus that forms a partially disordered strand, and a shorter C-terminus that completes the fold of domain I (Fig 2, Fig 3A). Structural alignment of PaMurB with EcMurB (type I), SaMurB (type IIa) and TcMurB (type IIb) highlights the presence of the β12-α5 loop and the consequential displacement of α5, and the protruding βαββ fold (β14-α7-β15-β16), which are not observed in type IIa/IIb enzymes (Fig 3B, Fig 2).

### Conserved FAD binding in PaMurB

PaMurB, FAD and NADP^+^ form a ternary complex at a 1∶1∶1 ratio. The FAD cofactor has well-defined electron density, occupying the same binding site in a conformation identical to the one previously reported in other MurB structures [8]–[10], [13], [15], [37] (Fig S2 and Fig S3). FAD is stabilized by multiple hydrogen bonds and van der Waals interactions with protein main chain and side chain atoms in the cofactor site. The binding site residues are essentially identical to those observed in the EcMurB structure (Fig S2). The isoalloxazine ring system is planar, suggesting that FAD is in its oxidized form. The yellow protein was immediately bleached upon addition of NADPH but recovered the yellow color, suggesting that reduction by NADPH is reversible under aerobic conditions.

### NADP(H) and UNAGEP share the same binding site

**Figure 4:**
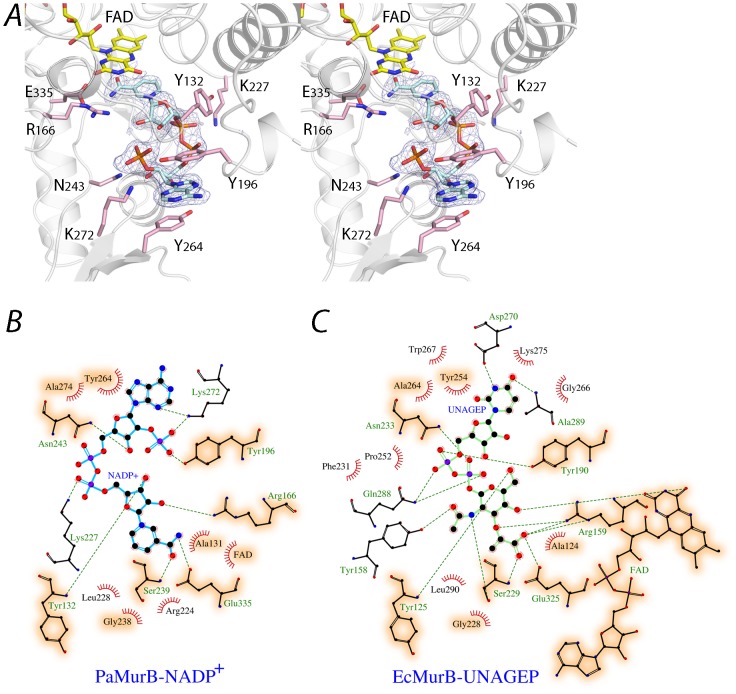
NADP^+^ and the substrate binding site. **A**. Stereo view of NADP^+^ bound in the substrate channel of PaMurB. NADP^+^ is depicted as a cyan stick model. The F_o_-F_c_ omit electron density of NADP^+^, contoured at 3.0 σ, indicates tight binding of the ligand. The nicotinamide ring stacks against the isoalloxazine ring system of FAD (shown in yellow). Residues of the binding site that form hydrogen bonds with NADP^+^ include Tyr-132, Arg-166 and Glu-335 for the nicotinamide moiety, Lys-227 for the diphosphate backbone, and Tyr-196, Asn-243 and Lys-272 for the adenosine. The adenosine moiety is in addition stabilized by stacking interactions with Tyr-196 and Tyr-264. **B and C**. Comparison of protein residues interacting with NADP^+^ and UNAGEP (based on EcMurB, PDB code 2MBR). NADP^+^ and UNAGEP are shown as ball-and-stick models in cyan and green, respectively. Red radiating lines around ligand atoms indicate van der Waals contacts, while green dashes represent potential hydrogen bonds. Ball-and-stick models are shown for binding site residues that provide polar interactions but not those involved in van der Waals interactions only. Orange shading highlights binding site residues that are conserved and involved in binding both substrates.

In the substrate binding cleft in domain III strong continuous electron density was observed that corresponds well to a bound NADP^+^ molecule (Fig 4A). NADP^+^ displays an extended “S” conformation where the 2-hydroxyl group of the nicotinamide ribose moiety interacts with the 2′-phosphate group of adenosine. The nicotinamide amide group is located on the *si* face of the isoalloxazine ring of FAD, and forms hydrogen bonds with the conserved Ser-239 and Glu-335 residues. The two rings are nearly parallel and juxtaposed together, with the nicotinamide C4n atom in close proximity to the isoalloxazine N5 atom (3.1 Å and 2.8 Å in crystal forms A and B, respectively). This suggests that the nicotinamide group is in a position that favors the first half-reaction (Fig 1). The phosphate-ribose backbone is stabilized by various active site residues and water bridges. Tyr-132 and Arg-166 interact with the nicotinamide ribose, while the phosphate backbone is stabilized by Lys-227 (Fig 4B). The phosphoribose in the adenosine moiety forms hydrogen bonds with Tyr-196, Asn-243 and Lys-272. The adenosine moiety, which is located at the entrance of the substrate channel, adopts the *anti* conformation with the adenine ring forming π-π stacking interactions with Tyr-196 and Tyr-264 from lobe 1 and the βαββ outcrop of lobe 2, respectively (Fig 4A&B).

**Figure 5:**
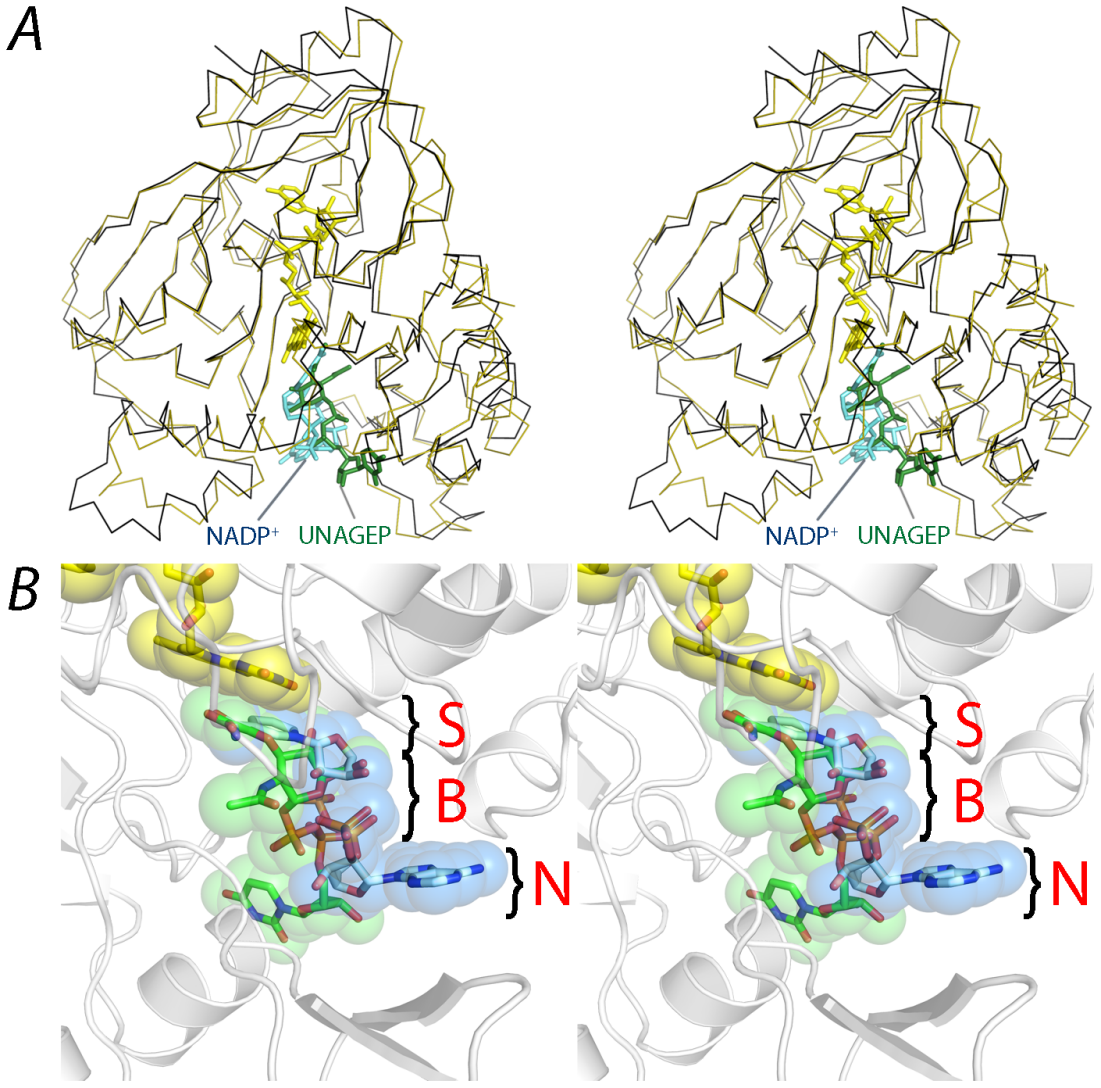
NADP^+^ shares the same substrate binding site with UNAGEP. **A**. Stereo view of PaMurB (black ribbon) and UNAGEP-bound EcMurB (olive green ribbon, 2MBR) superimposed based on their FAD atomic coordinates. FAD, NADP^+^ and UNAGEP are depicted as stick models in yellow, cyan and green, respectively. NADP and UNAGEP occupy the same substrate channel located between the lobes of domain III. **B**. Co-localization of NADP(H) and UNAGEP substrate moieties on the *si* face of the FAD isoalloxazine ring system. The reactive moieties of the two substrates align together after superimposing the structures of PaMurB-NADP^+^ and EcMurB-UNAGEP (PDB code 2MBR) based on the FAD atomic coordinates. However, the non-reactive parts of the ligands diverge in the binding site, which can be visualized as three loci. S: the substrate moiety that reacts with FAD. B: the backbone region consisting of sugar and diphosphate. N: the non-reactive nucleotide moiety, which shows the greatest deviation. The remodeling of the binding site according to individual substrates is discussed in the text.

Superimposition of the structure of the PaMurB ternary complex with UNAGEP-bound EcMurB (PDB code: 2MBR; rmsd 0.19 Å for all FAD atoms) reveals that NADP^+^ occupies the same binding site as UNAGEP, between the two lobes of the substrate-binding domain III (Fig 5A). The nicotinamide moiety occupies the position of the enolpyruvyl group of UNAGEP. The C4n atom of the nicotinamide ring, which is oxidized by FAD in the first half-reaction, is approximately aligned with C3e of the enolpyruvyl which in turn would receive the hydride from FADH_2_ during the second half-reaction (Fig 1). This is thus an overlapping sub-site for both substrates. However, the positions of the two molecules diverge at the pyrophosphate backbone and the non-reactive nucleotide moieties point in opposite directions (Fig 5B). Although the overall protein structure of the two complexes is preserved, the divergent binding of the two substrates causes structural changes in the PaMurB domain III; compared to the EcMurB-UNAGEP complex, the two lobes of domain III move away from the binding cleft. An analysis on domain movements using the program DynDom [38], [39] also reveals bending and rotational motions of the Tyr-196 loop and helices 5 and 6, as NADP^+^ binds closer to this lobe. Tyr-196 adopts a different rotamer, stabilizing NADP^+^ through a hydrogen bond with the adenosyl 2′-phosphate, and stacking interactions with the NADP^+^ adenine ring and Tyr-264 (Fig 4A). In addition, Lys-272 rotates inwards to form hydrogen bonds with the nicotinamide ring. Gln-288, which interacts with the diphosphate backbone of UNAGEP, adopts a different conformation and does not contact NADP^+^ because of the divergent binding mode (Fig 4C). A significant change in van der Waals contacts is also observed (Fig 4B&C). Although the non-reactive moieties do not overlap completely, the substrate channel formed by the two lobes of domain III can be visualized as three loci or subsites arranged from the active site interior to the channel opening (Fig 5B). Each locus is respectively occupied by the substrate moiety, pyrophosphate backbone and the nucleotide portion of either NADP(H) or UNAGEP.

### PaMurB contains a potassium ion in the active site

**Figure 6:**
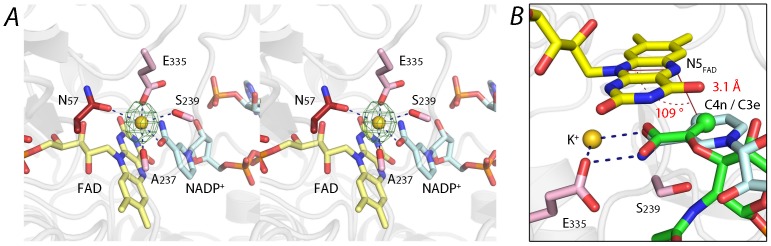
Potassium binding site in PaMurB and its proposed role in catalysis. **A**. Structure of the potassium binding site in PaMurB crystal form A. The coordination sphere is formed by the carboxamide oxygen of NADP^+^ nicotinamide in addition to two side chain oxygens and two main chain oxygens from the protein. The potassium ion (gold sphere) and its F_o_-F_c_ omit difference density contoured at 5.0 σ (green mesh) are shown. **B**. The active site potassium ion assists in substrate orientation and binding. Superimposition of the PaMurB crystal form A structure and the EcMurB-UNAGEP complex (PDB code 2MBR) based on FAD atomic coordinates shows that the C2-C3-C4 locus of NADP^+^ nicotinamide (in cyan) spatially overlap with the enolpyruvyl group of UNAGEP (in green). Both substrate moieties are bound to Glu-335 and the backbone amine of Ser-239. The nicotinamide C4n atom (cyan sphere), which transfers a hydride to the isoalloxazine N5 atom, coincides with the enolpyruvyl C3e (green sphere), which receives the hydride during the second half-reaction. The geometric relation of the C4n atom to the isoalloxazine is indicated. In synergy with the isoalloxazine ring, Glu-335 and Ser-239, the potassium ion positions NADPH and UNAGEP such that the substrate carbons are in the optimal position for hydride transfer.

A strong residual electron density in the vicinity of the nicotinamide ring was modeled as a metal ion in crystal form A (Fig 6A). The identity of the cation was inferred by alternative rounds of refinement using a water molecule, a sodium or potassium ion (present in the mother liquor) and a nickel ion (possibly derived from the Ni-NTA affinity chromatography step) as possible ligand. Refinement with potassium gave the most satisfactory results in terms of B factors (Table S1.) and difference densities. Sodium and water were ruled out as a possibility because of the abnormally low B factors after refinement and in the case of sodium also the mismatch in ligand-metal distances [40]. The metal site is located at the active site in the vicinity of the nicotinamide and the isoalloxazine rings of NADP^+^ and FAD, respectively. The potassium ion displays a pentagonal bipyramidal coordination geometry with two main chain oxygen atoms (Ala-237, Ser-239), one side chain carboxyl oxygen atom from Glu-335, one side chain oxygen atom of Asn-57 and the O7n oxygen atom of the nicotinamide ring as ligands. The interactions between Glu-335, nicotinamide and the metal ion position the nicotinamide C4n 3.1 Å from the isoalloxazine N5, and 109.3° from the isoalloxazine plane (defined as the C4n-N5-N10 angle) (Fig 6B). A strikingly close alignment between the enolpyruvyl from EcMurB and the nicotinamide is also observed upon superimposing the two structures, with a positional difference of 0.4 Å between the reactive C3e and C4n atoms. Crystal form B also shows electron density at this position, but refinement suggests that this site is occupied by a water molecule or a metal ion at lower occupancy (Table S1). Potassium binding apparent in only crystal form A could be due to the five times higher potassium concentration in the crystallization liquor for form A compared to form B crystals. It is noteworthy that the potassium concentration used to grow crystal form A is closer to physiological concentrations of this metal ion in the bacterial cell [41] than the significantly lower concentration in form B crystals.

## Discussion

### Hydride transfer occurs via the isoalloxazine si face

The crystal structure of the PaMurB- NADP^+^ complex presented in this work allows the comparison of NADP(H)- and UNAGEP-bound MurB structures. As proposed based on NMR [16], [17] and kinetic studies [7], the binding of NADPH and UNAGEP to MurB occurs in the same binding site and is mutually exclusive. The nicotinamide ring of NADP(H) occupies the same position as the enolpyruvyl group of UNAGEP, on the *si* face of the FAD isoalloxazine ring (Fig 5, Fig 6B). In the PaMurB ternary complexes, the nicotinamide C4n atom is located close to the N5 atom of the isoalloxazine ring (2.8–3.1 Å) and the 4-*pro*-S hydrogen atom of the *sp^3^*-hybridized C4n atom would point towards the N5 atom receiving the hydride ion (Fig 6B). The co-linear alignment of C4n, the 4-*pro*-S hydrogen and the isoalloxazine N5 atoms facilitates hydride transfer in the first half-reaction of the enzyme, the reduction of FAD. Thus, the conformation of NADP^+^ we observed most likely resembles the actual reactive complex. The UNAGEP enolpyruvyl C3e atom in the MurB-UNAGEP complex occupies the same position as the nicotinamide C4n atom, suitable for the hydride transfer in the second half-reaction. The flexibility of the substrate-binding domain accommodates the distinct substrates enabling hydride transfer to the isoalloxazine (Fig 4, Fig 5), without any requirement for significant re-arrangements of FAD, which might increase exposure of the hydride to bulk solvent. The difficulty in the previous NMR study to assign residues in the substrate binding site may reflect this dynamic nature [17]. As seen in EcMurB, Arg-166 and Arg-224 interact with the N1-C2-O2 and the N5 loci of isoalloxazine, a common feature of flavoenzymes thought to stabilize the reduced FAD [42].

The NADPH-FAD stereochemistry utilizing the isoalloxazine *si* face is rare and has only one known precedent, the NADH-dependent methylenetetrahydrofolate reductase (MetTHFR) that also employs the ping pong mechanism [43], [44]. Comparison of NADH- and methylenetetrahydrofolate-bound MetTHFR crystal structures (PDB codes 1ZPT and 1ZP4, respectively) reveals sharing of the substrate-binding cleft and alignment of hydride donor and acceptor substrate atoms, similar to the MurB-substrate complexes, despite having a different nicotinamide orientation relative to the FAD cofactor and a different NADH conformation.

### Potassium activates MurB by facilitating substrate orientation and binding

Monovalent cations have been shown to activate MurB [45], and potassium increased MurB activity more efficiently compared to other alkali metal ions and ammonium [7]. The K_m_ for both NADPH and UNAGEP was lowered and k_cat_ was increased with increasing potassium concentration. Hill plot analysis also suggested that one potassium ion is involved for each active site [45]. The observation of a bound potassium ion in the crystal structure of PaMurB (Fig 6A) provides a structural and chemical explanation for these findings. Superposition of the structure of crystal form A of PaMurB with the coordinates of the EcMurB-UNAGEP complex (PDB code 2MBR) shows that the carboxyl group of the substrate would be in coordination distance to the potassium ion. In the seminal paper by Hogle and colleagues [15] it was also proposed that the water molecule (W624 in PDB file 2MBR) observed at this position in the *E. coli* enzyme could potentially represent a metal site. Together with the conserved active site residues Ser-239 and Glu-335, which interact with both NADP^+^ nicotinamide and UNAGEP enolpyruvyl (Fig 4), the potassium ion orientates both substrate groups to facilitate MurB catalysis. Direct coordination of the enolpyruvyl group of the UNAGEP substrate would contribute to transition state stabilization during hydride transfer from reduced FAD to the substrate and thus explain the activation of the enzyme by monovalent ions. Also, the first step of the reaction, the reduction of FAD by NADPH is influenced by the lowered K_m_ for the co-substrate NADPH observed in the presence of potassium ions [7], [45], possibly due to the direct coordination of the nicotinamide carboxamide group to the metal ion.

### Discrimination against NADH is mediated by Tyr-196 and Lys-272

NADP(H) differs from NAD(H) only by the substitution of the adenosine 2′-OH by 2′-phosphate. Despite the similarity, conversion of UNAGEP to UNAM is undetectable in the presence of NADH [7]. In the structure of the ternary complex of PaMurB, Lys-272 forms hydrogen bonds with the 2′-phosphate and the N3 atom of the adenine moiety (Fig 4). Furthermore, in addition to stabilizing the adenine ring by stacking interactions, Tyr-196 also points towards the 2′-phosphate and forms a hydrogen bond through the phenolic hydroxyl group. These interactions may be important for achieving a catalytically competent ternary complex and orientating the nicotinamide to the correct catalytic site location. NADPH analogs lacking 2′- or 3′-phosphate i.e. 2′,3′-cyclic NADP^+^, NAD^+^ and NADH were unable to bind as substrates or inhibitors, while 3′-NADPH was an inefficient substrate [7], presumably because of the loss of stabilizing interactions with Tyr-196 and Lys-272.

## Conclusions

The crystal structure of the ternary complex of PaMurB-FAD-NADP^+^ provides direct evidence of overlapping substrate binding sites for NADPH and UNAGEP. Hydride transfer occurs to and from the *si* face of the FAD isoalloxazine ring system, with the donor carbon atom C4n of NADPH and the acceptor carbon atom C3e of UNAGEP in the same position, approximately 3 Å from the N5 atom of the isoalloxazine ring of FAD. A potassium ion in the active site is proposed to assist in substrate binding and stabilization of the UNAGEP transition state. Several important binding site residues are also found to provide the basis for substrate discrimination against NADH. Importantly, a three-fragment strategy targeting three loci in the substrate channel should prove invaluable in designing new inhibitors against MurB from *P. aeruginosa* as has been demonstrated for EcMurB [46].

## Supporting Information

Figure S1 PaMurB elutes as a single monomeric species in size-exclusion chromatography. Purified PaMurB was loaded on the Superdex 200 10/300 column (GE Healthcare) equilibrated with the protein buffer (25 mM Tris-Cl pH 8.0, 150 mM NaCl). The protein elutes as a single peak at 16.5 ml volume. The molecular mass of PaMurB calculated based on the calibration curve (insert) is 37.5 kDa, that agrees well with the value calculated from the sequence, 37.7 kDa.
(TIF)

Figure S2 Conservation of the MurB FAD-binding site. FAD binds to PaMurB and EcMurB (PDB code 2MBR) in an identical conformation, involving highly conserved hydrogen bonding and van der Waals contacts between the two proteins. FAD molecules are shown as purple ball-and-stick models. Hydrogen bonds and van der Waals interactions are indicated as green dashes and red radiating lines, respectively. Conserved interacting partners are circled.
(TIF)

Figure S3 F_o_-F_c_ omit electron density of FAD. FAD and NADP^+^ are depicted as stick models in yellow and cyan, respectively. The electron density is contoured at 3.0 σ and depicted as a green mesh.
(TIF)

Table S1 Analysis of the putative metal site in PaMurB. A water molecule or one of the three different metal ions, Na^+^, K^+^ or Ni^2+^ were tested to model the residual electron density near the nicotinamide carboxamide group of NADP+. All metal ions were refined with their appropriate charges specified. Comparison of the resulting atomic B factor with that of coordinating oxygen atoms and donor-acceptor distances suggest that potassium binds to the site in crystal form A.
(DOCX)
