# Supplementary figures and images for: Substrate Channel Flexibility in Pseudomonas aeruginosa MurB Accommodates Two Distinct Substrates
Source: PLoS One. 2013 Jun 21;8(6):e66936. doi: 10.1371/journal.pone.0066936 (PMC3689657; doi:10.1371/journal.pone.0066936)

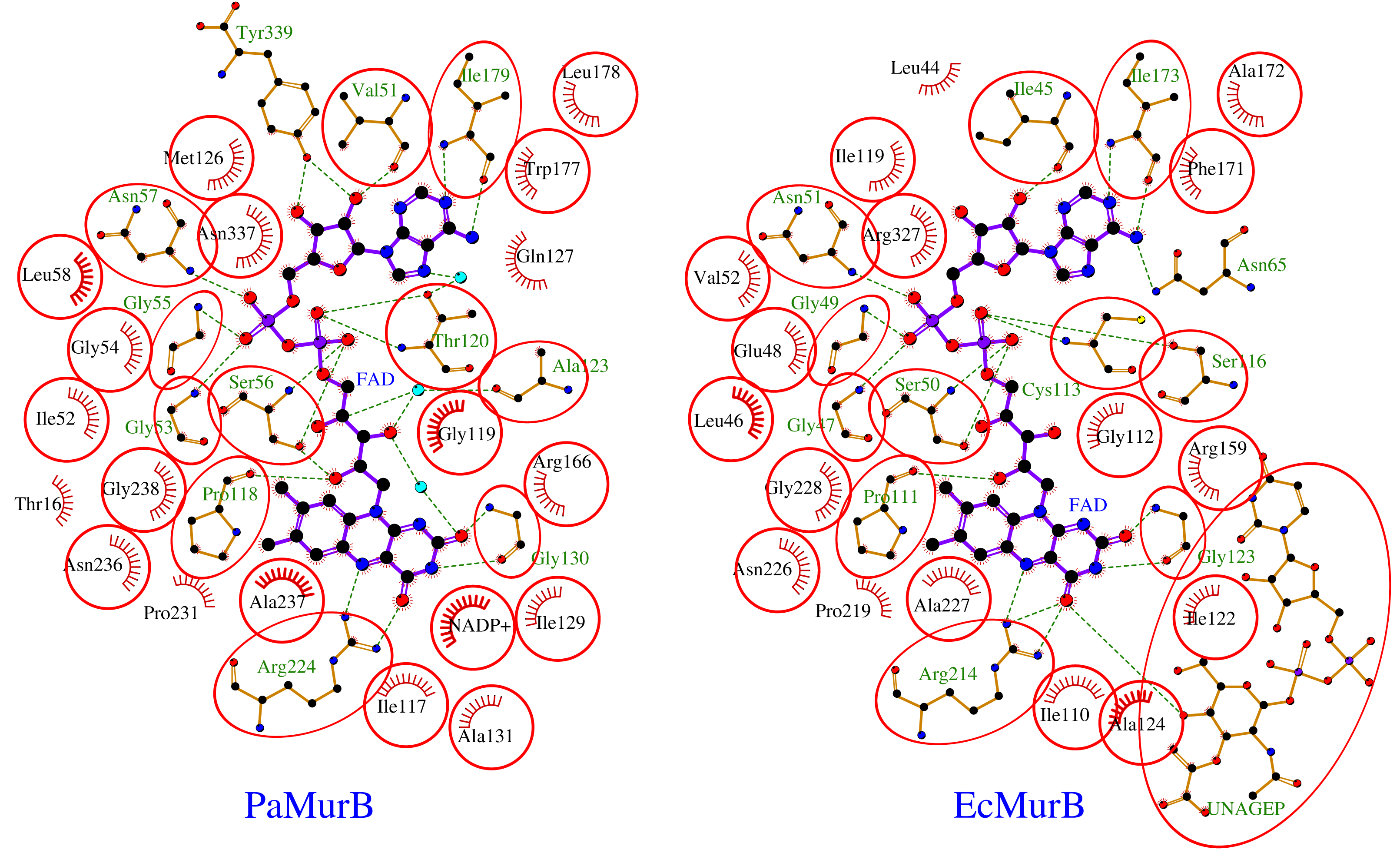

Supplement: Figure S2 — Conservation of the MurB FAD-binding site. FAD binds to PaMurB and EcMurB (PDB code 2MBR) in an identical conformation, involving highly conserved hydrogen bonding and van der Waals contacts between the two proteins. FAD molecules are shown as purple ball-and-stick models. Hydrogen bonds and van der Waals interactions are indicated as green dashes and red radiating lines, respectively. Conserved interacting partners are circled. (TIF) [file pone.0066936.s002.tif]

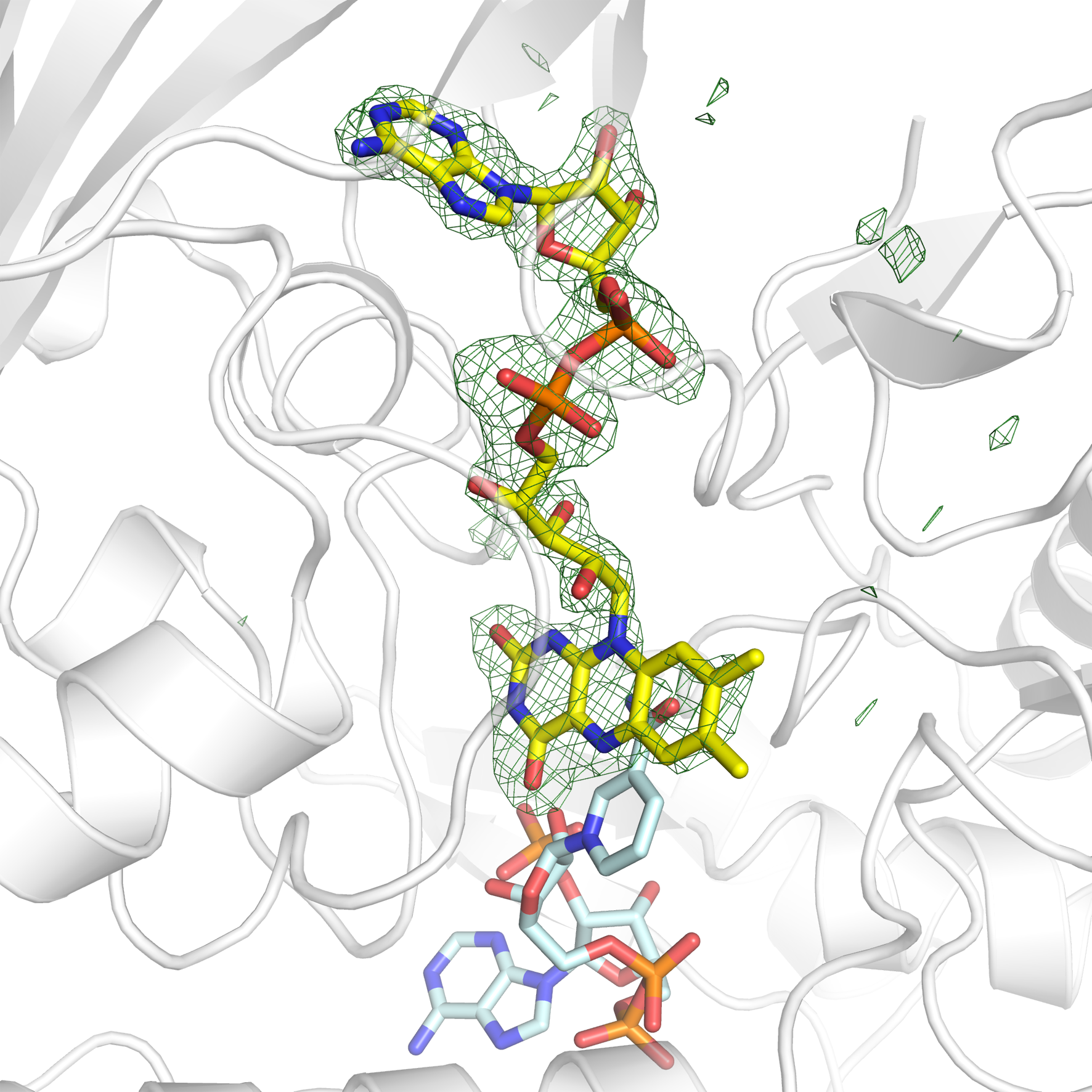

Supplement: Figure S3 — Fo-Fc omit electron density of FAD. FAD and NADP+ are depicted as stick models in yellow and cyan, respectively. The electron density is contoured at 3.0 σ and depicted as a green mesh. (TIF) [file pone.0066936.s003.tif]
