# Supplementary material for: Substrate Channel Flexibility in Pseudomonas aeruginosa MurB Accommodates Two Distinct Substrates
Source: PLoS One. 2013 Jun 21;8(6):e66936. doi: 10.1371/journal.pone.0066936 (PMC3689657; doi:10.1371/journal.pone.0066936)
Supplement: Table S1 — Analysis of the putative metal site in PaMurB. A water molecule or one of the three different metal ions, Na+, K+ or Ni2+ were tested to model the residual electron density near the nicotinamide carboxamide group of NADP+. All metal ions were refined with their appropriate charges specified. Comparison of the resulting atomic B factor with that of coordinating oxygen atoms and donor-acceptor distances suggest that potassium binds to the site in crystal form A. (DOCX) [file pone.0066936.s004.docx]

**Table S1.**

| Crystal form | Atomic B factor of model atom at occ=1.0 (Å^2^) | | | | Atomic B factor  of ligand atom* (Å^2^) | Metal-ligand  distance* (Å) |
| --- | --- | --- | --- | --- | --- | --- |
|  | H_2_O | Na^+^ | K^+^ | Ni^2+^ |  |  |
| A (4 mol/asu) | 2.3,  2.0,  2.0,  2.6 | 5.3,  3.8,  5.6,  6.1 | 20.0,  16.3,  19.7,  19.9 | 37.7,  33.1,  36.8,  36.7 | Oδ_N57_ = 22.4  O_A237_ = 17.8  O_S239_ = 19.1  Oε_E335_ = 37.1  O7_NADP_ = 17.3 | Oδ_N57_ = 2.7  O_A237_ = 2.7  O_S239_ = 2.7  Oε_E335_ = 2.3  O7_NADP_ = 3.0 |
| B (1 mol/asu) | 27.3 | 39.4 | 62.1 | - | Oδ_N57_ = 26.0  O_A237_ = 29.3  O_S239_ = 27.4  Oε_E335_ = 31.6 | Oδ_N57_ = 2.8  O_A237_ = 2.7  O_S239_ = 2.9  Oε_E335_ = 2.3 |

* averaged value of 4 NCS copies in the case of crystal form A.
